# A simple synthesis of transparent and highly conducting p-type Cu_*x*_Al_1−*x*_S_*y*_ nanocomposite thin films as the hole transporting layer for organic solar cells†

**DOI:** 10.1039/c8ra01299g

**Published:** 2018-05-08

**Authors:** Xin Dai, Hongwei Lei, Cong Chen, Yaxiong Guo, Guojia Fang

**Affiliations:** Key Laboratory of Artificial Micro- and Nano-structures of Ministry of Education of China, School of Physics and Technology, Wuhan University Wuhan 430072 PR China; Shenzhen Institute of Wuhan University Shenzhen 518055 PR China gjfang@whu.edu.cn +86 (0)27 68752569 +86 (0)27 87642784

## Abstract

Inorganic p-type films with high mobility are very important for opto-electronic applications. It is very difficult to synthesize p-type films with a wider, tunable band gap energy and suitable band energy levels. In this research, p-type copper aluminum sulfide (Cu_*x*_Al_1−*x*_S_*y*_) films with tunable optical band gap, carrier density, hole mobility and conductivity were first synthesized using a simple, low cost and low temperature chemical bath deposition method. These *in situ* fabricated Cu_*x*_Al_1−*x*_S_*y*_ films were deposited at 60 °C using an aqueous solution of copper(ii) chloride dihydrate (CuCl_2_·2H_2_O), aluminium nitrate nonohydrate [Al(NO_3_)_3_·9H_2_O], thiourea [(NH_2_)_2_CS], and ammonium hydroxide, with citric acid as the complexing agent. Upon varying the ratio of the precursor, the band gap of the Cu_*x*_Al_1−*x*_S_*y*_ films can be tuned from 2.63 eV to 4.01 eV. The highest hole mobility obtained was 1.52 cm^2^ V^−1^ s^−1^ and the best conductivity obtained was 546 S cm^−1^. The Cu_*x*_Al_1−*x*_S_*y*_ films were used as a hole transporting layer (HTL) in organic solar cells (OSCs), and a good performance of the OSCs was demonstrated using the Cu_*x*_Al_1−*x*_S_*y*_ films as the HTL. These results demonstrate the remarkable potential of Cu_*x*_Al_1−*x*_S_*y*_ as hole transport material for opto-electronic devices.

## Abstract 2

Inorganic p-type films with high mobility are very important for opto-electronic applications.

## Graphical abstract

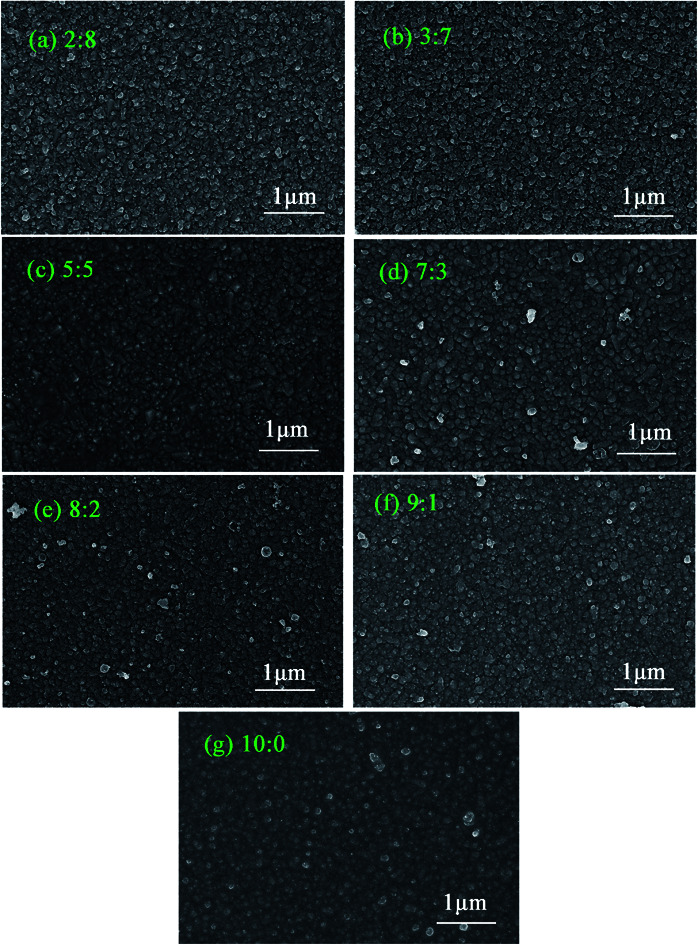

## 1. Introduction

Industrialization in the past years has yielded an increasing energy demand, which was resolved using non-renewable resources. However, utilization of these resources results in serious environmental pollution and depletion of the fossil fuel energy. To avoid this green renewable energy must be developed and solar cells are one of the most important ways to overcome this problem.^1–3^ Scientists have devoted much time to developing better carrier transporting layers with excellent properties.^4–6^ At present, organic solar cells (OSCs) have attracted much attention because of their light weight, flexibility, ease of production and high efficiency.^7–12^ Poly(3,4-ethylenedioxythiophene):poly(styrenesulfonate) (PEDOT:PSS)^13,14^ is a widely used hole transporting layer (HTL) which is used to modify the anode interface to improve the hole collection ability. However, because of its high hygroscopicity and acidity,^15^ PEDOT:PSS has an adverse impact on the device stability. To solve this problem, several materials with high transmissivity within the range of visible light have been used as alternatives, and these are materials such as molybdenum oxide (MoOx),^16–18^ nickel oxide (NiOx)^19,20^ and so on. However, these materials are either toxic or in short supply. In addition, some of them often require complex vacuum systems for deposition. Therefore, it is highly desirable to develop a highly transparent, earth-abundant, low-cost, non-toxic and non-corrosive HTL for highly efficient OSCs and other opto-electronic applications. Inorganic p-type films with high mobility are very promising for opto-electronic applications. Nevertheless, it is difficult to synthesize p-type films with wider, tunable band gaps and suitable band energy levels.

Chalcogenide (CuAlS_2_) which is known to be a p-type semiconductor, is a promising material because of its high band gap energy (*E*_g_) and hole conductivity.^21,22^ There are various methods used to prepare CuAlS_2_, such as chemical vapor transport deposition, solid phase reaction, solvothermal deposition, chemical spray pyrolysis, hydrothermal methods and chemical bath deposition (CBD).^23–28^ However, these methods involve either high temperature or high pressure and the prepared CuAlS_2_ always has a low conductivity, whereas the film prepared using CBD is not very uniform and lacks measureable electrical properties.

In this research, a CBD technique was used to grow Cu_*x*_Al_1−*x*_S_*y*_ thin film with a tunable band gap *in situ*. Compared with other deposition methods,^23–27^ this method has several advantages such as: (1) the precursors are dissolved in distilled water, which is non-toxic, low cost and environmentally friendly, (2) the chemicals are commercially available and inexpensive, the deposition temperature is 60 °C which is really low and very safe, and (3) the process is simple. With this method Cu_*x*_Al_1−*x*_S_*y*_ films with a large area can easily be grown without using sophisticated instruments. In addition, citric acid and ammonium hydroxide are used to adjust the speed of the reaction to obtain the required film.

In comparison with copper(ii) sulfide (CuS)^29–31^ film, the Cu_*x*_Al_1−*x*_S_*y*_ film is easy to grow without needing any sophisticated instruments. Most importantly, the variable energy levels of Cu_*x*_Al_1−*x*_S_*y*_ films with a tunable band gap are very attractive. In this research, non-toxic and earth-abundant Cu_*x*_Al_1−*x*_S_*y*_ thin film was used as HTL for OSCs with a blend of poly(3-hexylthiophene) (P3HT) and 6,6-phenyl C_61_ butyric acid methyl ester (PCBM) was used as the active layer. Photovoltaic devices were made with the structure of fluorine doped tin oxide (FTO)/Cu_*x*_Al_1−*x*_S_*y*_/P3HT:PCBM/Al. The optimized OSCs illuminated under simulated AM1.5G, 100 mW cm^−2^ white light yielded a power conversion efficiency (PCE) of 2.67% with an open-circuit voltage (*V*_oc_) of 0.596 V, a short-circuit current density (*J*_sc_) of 9.21 mA cm^−2^, and a fill factor (FF) of 48.7%, which was comparable to that obtained with reference organic photovoltaics with PEDOT:PSS as HTL.

## 2. Experimental details

### 2.1 Material

The polymer donor (P3HT) was purchased from Rieke Metals and acceptor PC_61_BM was obtained from Nano-C. Copper(ii) chloride dihydrate (CuCl_2_·2H_2_O), aluminium nitrate nonohydrate [Al(NO_3_)_3_·9H_2_O], thiourea [(NH_2_)_2_CS], citric acid (all analytically pure reagents) and ammonium hydroxide were purchased from traditional Chinese chemical companies. All the materials were used without further purification. The solvent used was deionized water.

### 2.2 Preparation of Cu_*x*_Al_1−*x*_S_*y*_ films on FTO glass

The FTO substrate was cleaned using ultrasonic cleaning in deionized water, followed by acetone and alcohol for 10 min each. Finally, the substrates were dried in a temperature controlled drying oven.

Aqueous solutions of 0.01 M CuCl_2_·2H_2_O, 0.01 M Al(NO_3_)_3_·9H_2_O, 0.04 M (NH_2_)_2_CS, 0.02 M citric acid and pH adjuster (ammonium hydroxide) was used to prepare a CuAlS_2_ thin film. Firstly, CuCl_2_·2H_2_O, Al(NO_3_)_3_·9H_2_O and citric acid were placed into a beaker using deionized water as the solvent and then stirred continuously for a few minutes until the solution became homogenous. Then, ammonium hydroxide was added dropwise into the solution until it became a bluish violet colour, and solution was denoted as solution A. The (NH_2_)_2_CS was placed into another beaker and dissolved in deionized water, which was denoted as solution B. Solution A and solution B were mixed together to obtain the final solution, and ammonium hydroxide was added dropwise to adjust the pH to 8.8. Subsequently, the FTO substrates were placed into the final solution at 60 °C to obtain the required CuAlS_2_ film. To prepare the Cu_*x*_Al_1−*x*_S_*y*_ film (*x* = 0.2, 0.3, 0.5, 0.7, 0.8, 0.9, 1), the molar ratio of CuCl_2_·H_2_O and Al(NO_3_)_3_·9H_2_O was varied, while maintaining the total concentration at 0.02 M, as the remaining steps were processed as described previously. Then the Cu_*x*_Al_1−*x*_S_*y*_ films were annealed at 150 °C for 10 min and cooled down to room temperature (RT) prior to use.

### 2.3 Fabrication of solar cells

The organic solution was prepared by dissolving 10 mg of P3HT and 10 mg of PCBM in 0.5 ml of chlorobenzene with vigorous magnetic stirring for 24 h before use. The organic solution was used for the active layer deposition. It was spin-coated onto Cu_*x*_Al_1−*x*_S_*y*_ films at 500 rpm for 6 s and then at 1000 rpm for 20 s, to give a thickness of about 200 nm. The 100 nm thick Al top electrodes were thermally evaporated through a shadow mask under a pressure of about 10^−4^ Pa. Finally, the fabricated devices were thermally annealed on a hot plate at 150 °C for 10 min in an argon filled glovebox. The active area of the device was 0.04 cm^2^ as defined by the shadow mask.

### 2.4 Films and device characterization

The transmittance of the films was measured with an ultraviolet-visible -near infrared (UV-Vis-NIR) spectrophotometer (Cary 5000, Varian) in the 300–800 nm wavelength range at RT. The film thickness was measured using ellipsometry. Field-emission scanning electron microscopy (SEM; FEI XL-30) was used to observe the morphology of the samples. Transmission electron microscopy (TEM; Jeol JEM-2010) was used for the observation of the ultrastructure. Energy dispersive spectrometry (EDS; FEI XL-30) was used to determine the components of the samples. X-ray photoelectron spectroscopy (XPS) and ultraviolet photoelectron spectroscopy (UPS) were performed using a XPS/UPS system (ThermoScientific, ESCLAB 250Xi, USA). The compositions and chemical states of the CuAlS_2_ films were examined using XPS. Before being tested, the samples were sputter cleaned, to remove atmospheric contamination in the XPS chamber for approximately 30 s, using the lower energy of Ar^+^, and the Ar^+^ gun was operated at 0.5 kV under a pressure of 1 × 10^−7^ Pa. The vacuum pressure of the analysis chamber was greater than 1 × 10^−8^ Pa. A whole survey scan to identify the overall surface composition and chemical states was performed, using a monochrome Al Kα X-ray source (1486.68 eV), with detection of photoelectrons at a 150 eV energy pass and a channel width of 500 meV. The surface carbon signal at 284.6 eV was used as an internal standard. The work function and band energy levels were measured using UPS. UPS was carried out using helium *I*_α_ radiation from a discharge lamp operated at 90 W, a pass energy of 10 eV, and a channel width of 25 meV. A −9 V bias was applied to the samples, in order to separate the sample and determine the low kinetic energy cutoffs. The conductivity, carrier concentration and mobility were measured using a Hall effect measurement system (Lake Shore Cryotronics, 7704A). The current–voltage (*J*–*V*) curves of the devices were obtained using a computer controlled Source Measure Unit (Keithley 2400) and the device test was carried out in a glove box under illumination of AM1.5G, 100 mW cm^−2^ (the light intensity was calibrated using a silicon photodiode) at RT using a solar simulator.

## 3. Results and discussion

**Fig. 1:**
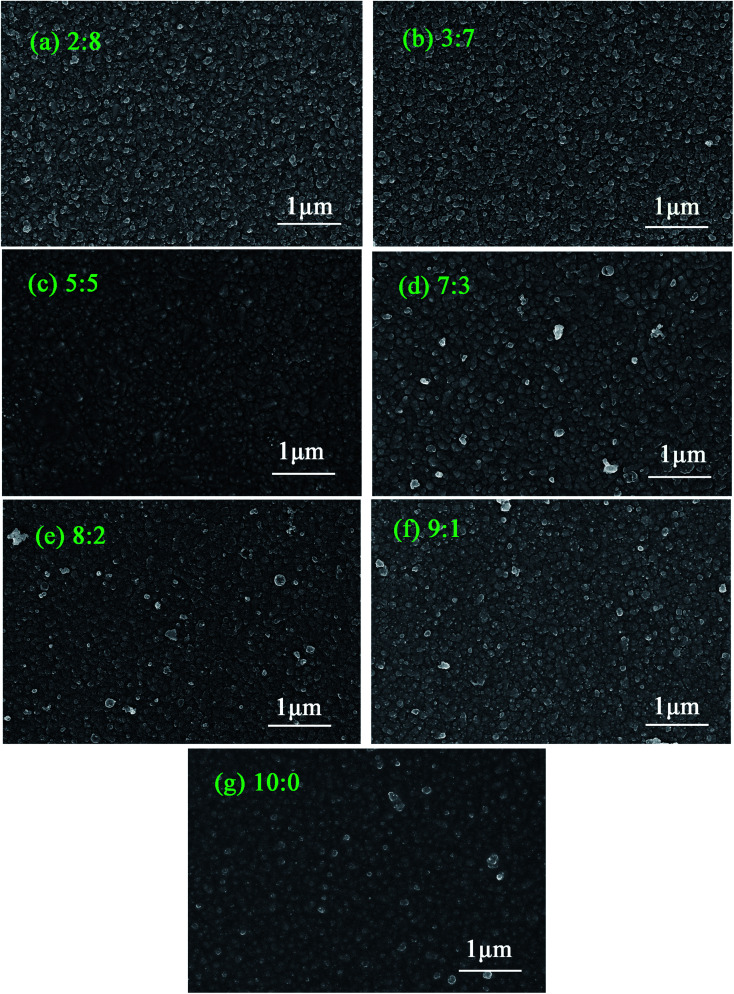
SEM images of Cu_*x*_Al_1−*x*_S_*y*_ films fabricated on FTO substrates. (a) *x* = 0.2, Cu:Al = 2 : 8. (b) *x* = 0.3, Cu:Al = 3 : 7. (c) *x* = 0.5, Cu:Al = 5 : 5. (d) *x* = 0.7, Cu:Al = 7 : 3. (e) *x* = 0.8, Cu:Al = 8 : 2. (f) *x* = 0.9, Cu:Al = 9 : 1. (g) *x* = 1, Cu:Al = 10 : 0.

The SEM images of Cu_*x*_Al_1−*x*_S_*y*_ thin films annealed at 150 °C for 10 min with various composition are shown in Fig. 1. The ratio shown in each SEM image is the ratio of Cu:Al which refers to *x* = 0.2, 0.3, 0.5, 0.7, 0.8, 0.9, or 1. The films deposited at 60 °C for 10 min were very uniform. The particle size was defined by testing several small particles using a scaleplate when magnifying the picture. The average size of the particles was about 50–100 nm. As observed, both a cluster-by-cluster process and an ion-by-ion process occurred in the reaction, and the ion-by-ion process was dominant in the formation which could be seen from the small crystal domains in the films. The CuAlS_2_ (5 : 5) and CuS (10 : 0) films show a similar, smoother film because of their structure, whereas the Cu_*x*_Al_1−*x*_S_*y*_ (8 : 2) film has similar round domains.

**Fig. 2:**
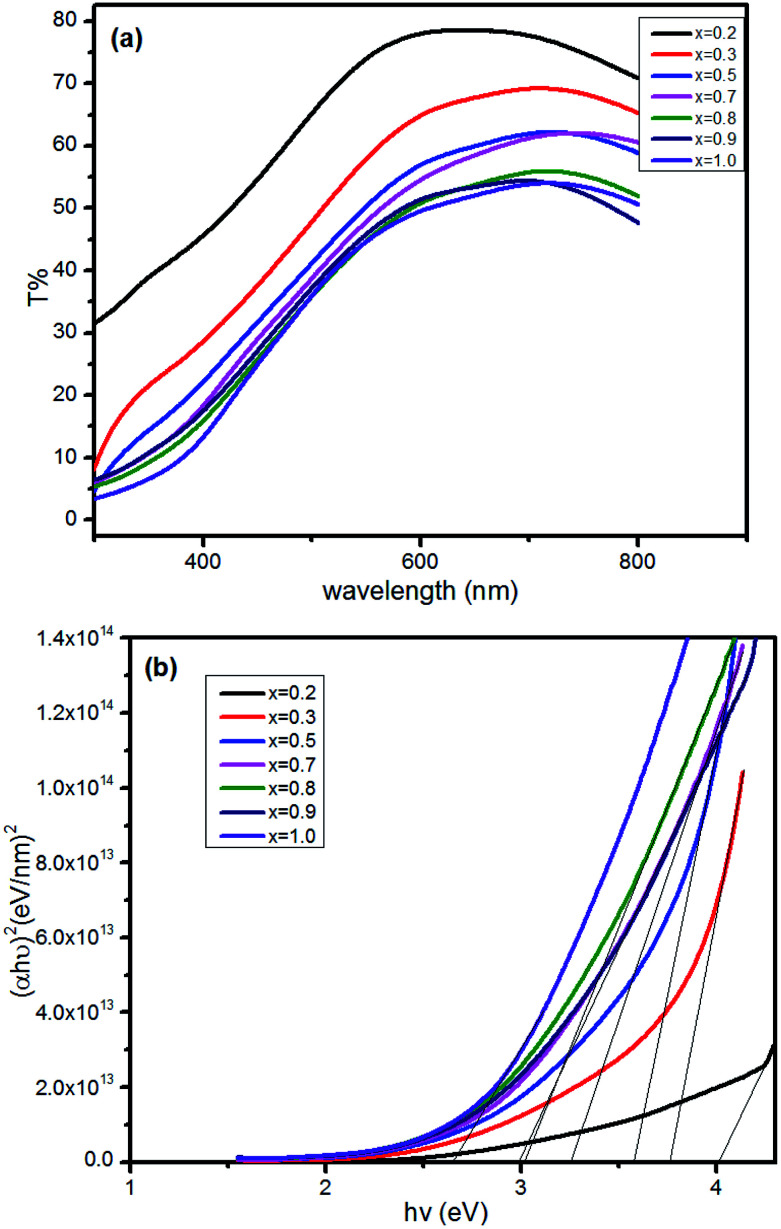
The transmission spectra of Cu_*x*_Al_1−*x*_S_*y*_ films deposited on glass after annealing at 150 °C for 10 min (a), and the diagram of (*αhν*)^2^ against *hν* calculated from the transmission spectra (b).

The transmission spectra of Cu_*x*_Al_1−*x*_S_*y*_ thin films are shown in Fig. 2(a) (*x* = 0.2, 0.3, 0.5, 0.7, 0.8, 0.9, 1). The film transmittance increased with Al content when the thickness of the films was approximately 200 nm. It should be noted that in this research, the film thicknesses were all tested using an ellipsometer.

The energy gap *E*_g_ was calculated from the equation:*αhν* = *B*(*hν* − *E*_g_)^*r*^where *α* is the absorption coefficient, which can be calculated from the equation: 
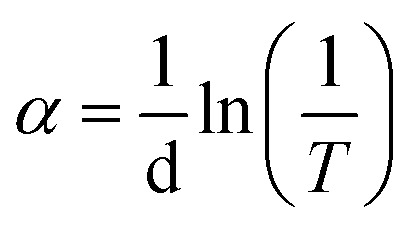
*B* is a constant and *r* is an index, which could have values of 1/2, 3/2, 2 and 3, depending on the nature of the electronic transition. The exponent *r* = 1/2 is for allowed direct transition, *r* = 3/2 is for forbidden direct transition, *r* = 2 is for forbidden indirect transition, and *r* = 3 is for allowed indirect transition.

The CuAlS_2_ film exhibited allowed direct transition. The optical band gap of Cu_*x*_Al_1−*x*_S_*y*_ film according to the dependence of (*αhν*)^2^ on *hν* was confirmed. The estimated band gaps of the Cu_*x*_Al_1−*x*_S_*y*_ films at different Cu concentrations are illustrated in Fig. 2(b). Using Fig. 2(b), the CuAlS_2_ film (*x* = 0.5) was calculated to have a band gap of 3.60 eV which matched well with those figures reported in the literature. Also, as expected, CuS film (*x* = 1) had an optical gap of 2.63 eV, which was very close to the reported values (2.4 eV). As can be seen in Fig. 2(b), starting from CuS, the band gap of the films increased from 2.63 eV to 4.01 eV with the decrease of *x* (which means that the ratios of Cu to Al became smaller) and the transmittance increased with the decreasing Cu content. Thus, metal chalcogenides with a tunable optical band gap could be obtained by varying the precursor ratio of Cu and Al, and this is promising for applications in optoelectronic devices.

**Fig. 3:**
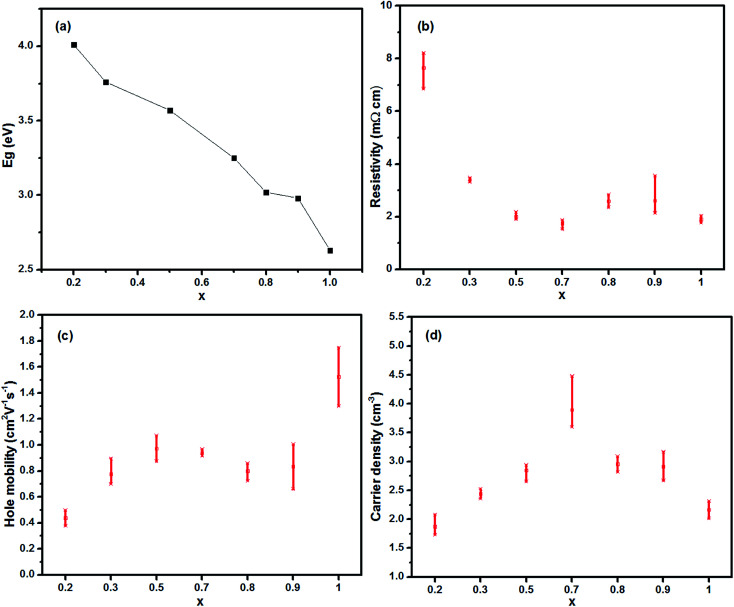
(a) Energy band gap, (b) resistivity, (c) hole mobility, (d) carrier density of the Cu_*x*_Al_1−*x*_S_*y*_ films at room temperature as a function of the Cu concentration.

The electrical properties were characterized using Hall effect measurements. As seen in Table 1, there was a variation of optical and electrical parameters of the Cu_*x*_Al_1−*x*_S_*y*_ thin films which were annealed at 150 °C for 10 min. For each composition with a particular molar ratio, more than three samples were made and tested to ensure the reproducibility, and all the films displayed p-type conductivity. The data listed in Table 1 for the reference was the one which was closer to the average result. Overall, the resistivity shows a tendency to decrease with increasing Cu concentration, as shown in Fig. 3(b). A maximum conductivity of 546 S cm^−1^ in films with *x* = 0.7 was achieved, which was much higher than the values reported for the p-type HTLs.^32,33^ It is interesting that the resistivity of the films between *x* = 0.7 and *x* = 0.8 has a sudden rise, the reason for this is not known at the moment. Hole concentration and mobility were measured using a 7704A Hall system (Lake Shore Cryotronics). As shown in Fig. 3(c), hole mobility appears to increase gradually as the Cu concentration increased, within the range of 0.2 < *x* < 0.5, and then gently decreased and this takes no account of the CuS film. Compared with other samples, the high conductivity of the film with *x* = 0.7 originates from the relatively higher carrier concentration and mobility. In Fig. 3(d), hole concentration varies from (1–4) × 10^21^ cm^−3^, which is in the range of a highly doped degenerate semiconductor.

**Table 1:** Summary of optical and electrical properties of the Cu_*x*_Al_1−*x*_S_*y*_ film annealed at 150 °C for 10 min

x	*E*_*g*_ (eV)	*R* (mΩ cm)	*R* (Ω sq^−1^)	Hall mobility (cm^2^ V^−1^ s^−1^)	Carrier density (cm^−3^)
0.2	4.01	7.88	342.50	0.44	1.81 × 10^21^
0.3	3.76	3.32	165.89	0.80	2.36 × 10^21^
0.5	3.60	2.18	109.16	0.97	2.94 × 10^21^
0.7	3.25	1.83	122.02	0.94	3.61 × 10^21^
0.8	3.02	2.84	113.41	0.78	2.82 × 10^21^
0.9	2.91	2.15	107.39	0.92	3.17 × 10^21^
1	2.63	2.04	88.48	1.52	2.01 × 10^21^

Fig. 3 reveals that the band gaps of 2.63–4.01 eV were comparable to p-type transparent materials such as aluminium copper dioxide (CuAlO_2_; 3.6 eV), and the hole conductivity was relatively high, which was ascribed to the considerable mobility of 0.4–1.5 cm^2^ V^−1^ s^−1^ and a hole concentration of 1−4 × 10^21^ cm^−3^. CuS is well known as a p-type conductor. Thus, the hole conduction in the Cu_*x*_Al_1−*x*_S_*y*_ films was attributed to the CuS phase. The conducting network formed by CuS (even in Al rich samples) leads to the high p-type conductivity in the films.

As discussed previously, transparency is more dependent on Cu and Al contents, whereas the Cu content plays an important role in the hole conductivity as well. Of the films with <1000 Ω sq^−1^ sheet resistance, except for *x* = 1, the highest transparency was found at *x* = 0.2 because it had the highest Al content whereas the lowest sheet resistance and the highest hole mobility was found at *x* = 0.5. The EDS measurements were used to determine the components of the Cu_*x*_Al_1−*x*_S_*y*_ films. As shown in Table 2, the atom percentage of Al decreased from 76.02% to 0% when *x* increased, which may be the main reason for the reduction of the optical band gap. In addition, the increasing atom percentage of Cu and S when *x* increases might be responsible for the increasing hole mobility. For the CuS film (*x* = 1), the mole ratio of Cu and S revealed the existence of copper sulfide (Cu_2_S) and CuS. The possible existence of the O element in the film may result in the higher energy gap (2.63 eV).

**Table 2:** Summary of the component analysis of the Cu_x_Al_1-x_S_y_ film characterized using EDS

x in precursor	Cu (atom%)	Al (atom%)	S (atom%)
0.2	23.50	76.02	0.48
0.3	23.8	52.9	23.3
0.5	47.17	22.04	30.8
0.7	54.46	14.32	33.22
0.8	53.62	11.34	35.04
0.9	58.78	6.9	34.32
1	61.12	0	38.88

**Fig. 4:**
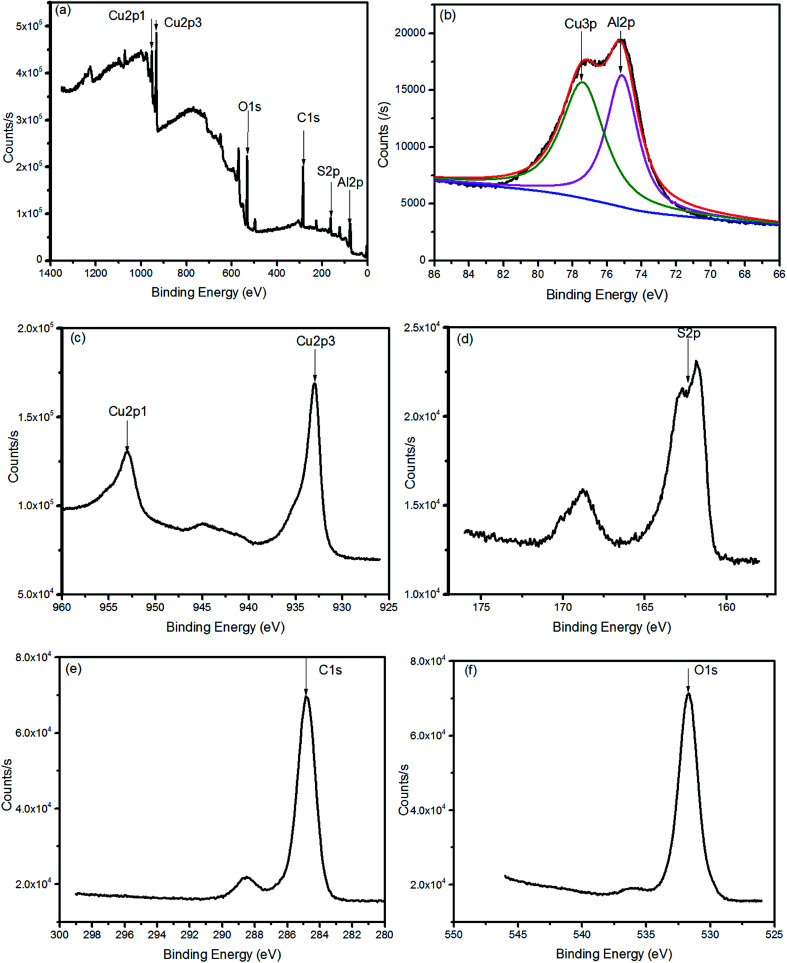
The XPS spectra of CuAlS_2_ films on FTO glass (a), (b), (c), (d), (e) and (f).

**Fig. 5:**
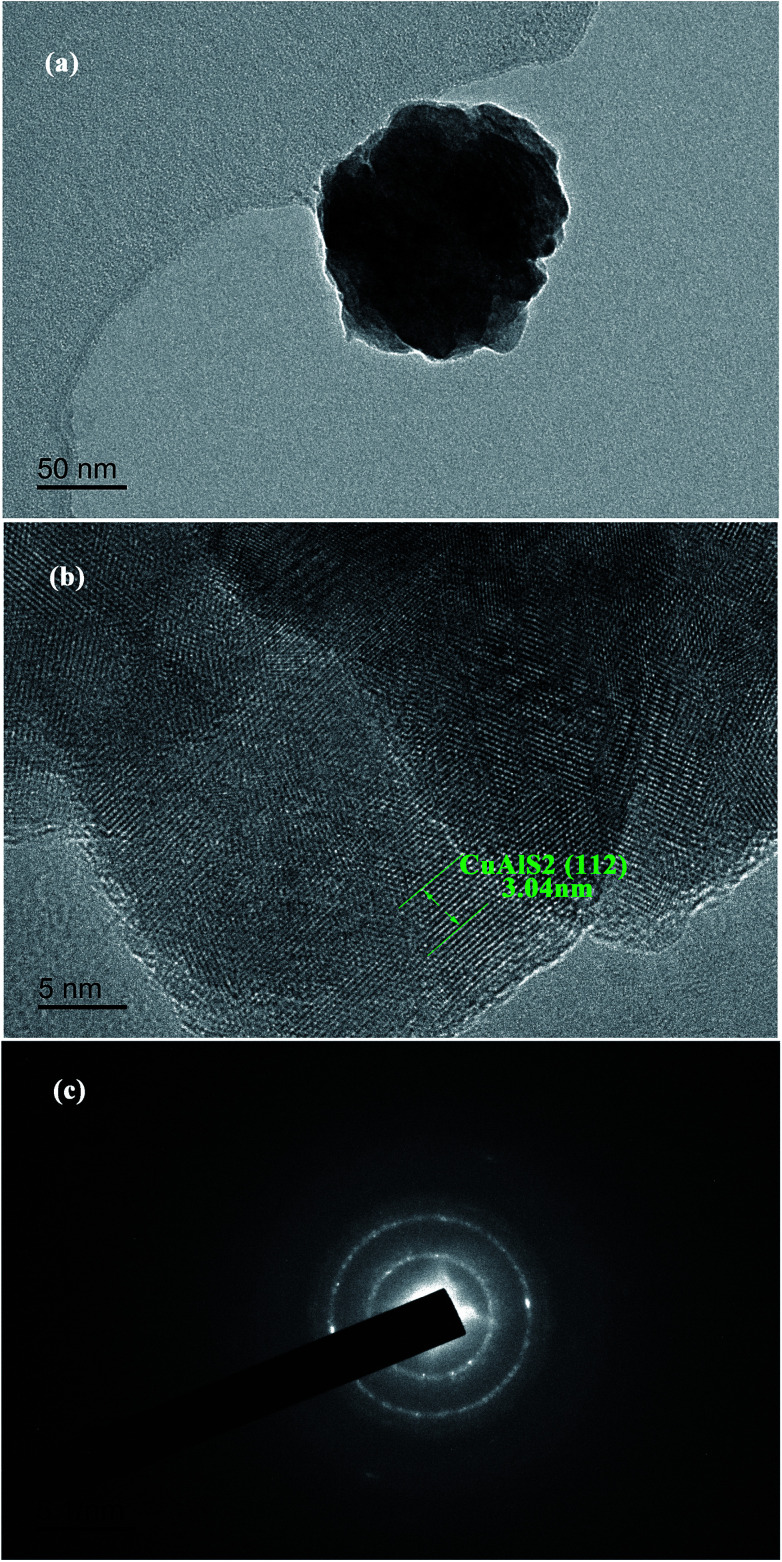
(a) TEM, (b) HRTEM images and (c) SAED pattern of CuAlS_2_.

The XPS spectra of CuAlS_2_ films (*x* = 0.5, annealed at 150 °C) deposited on glass are shown in Fig. 4. Fig. 4(a) shows a full scale scan of the results of the XPS which found the peaks of Cu, Al, O, S and C. The magnified peaks of the Al 2p, Cu 2p and S 2p scan are shown in Fig. 4(b), (c) and (d), respectively. Fig. 4(b) shows the peak fitting of the Al 2p spectra. The peak fitting for the Al 2p line was divided into two peaks, the peaks at 77 eV and 74 eV revealed the presence of Cu 3p_3/2_ and Al 2p. The peaks of Cu 2p were at 952.9 eV and 932.9 eV. The core levels of Cu 2p_1/2_ indicated that there was a divalent Cu ion in the product whereas the core level of Cu 2p_3/2_ refers to the Cu^+^.^34^ The peaks at 162.0 eV belonged to S 2p. These results were consistent with the results found in the literature and have proved that the atomic ratio of elemental Cu and Al was approximately 2 : 1. For further analysis of the CuAlS_2_ films, TEM measurements were made to observe the ultrastructure. As seen in Fig. 5(a), the particles seem very large which may be because of the thick film. Fig. 5(b) is a typical high resolution TEM (HRTEM) image of the film of CuAlS_2_, where 10 crystal planes were tested to get an average interplanar distance for each crystal orientation. It was concluded that there is CuAlS_2_ (112) in the CuAlS_2_ film with a corresponding interplanar distance of 3.04 Å. The selected area electron diffraction (SAED) pattern of the CuAlS_2_ film in Fig. 5(c) shows clearly that the CuAlS_2_ film is a polycrystalline compound.

**Fig. 6:**
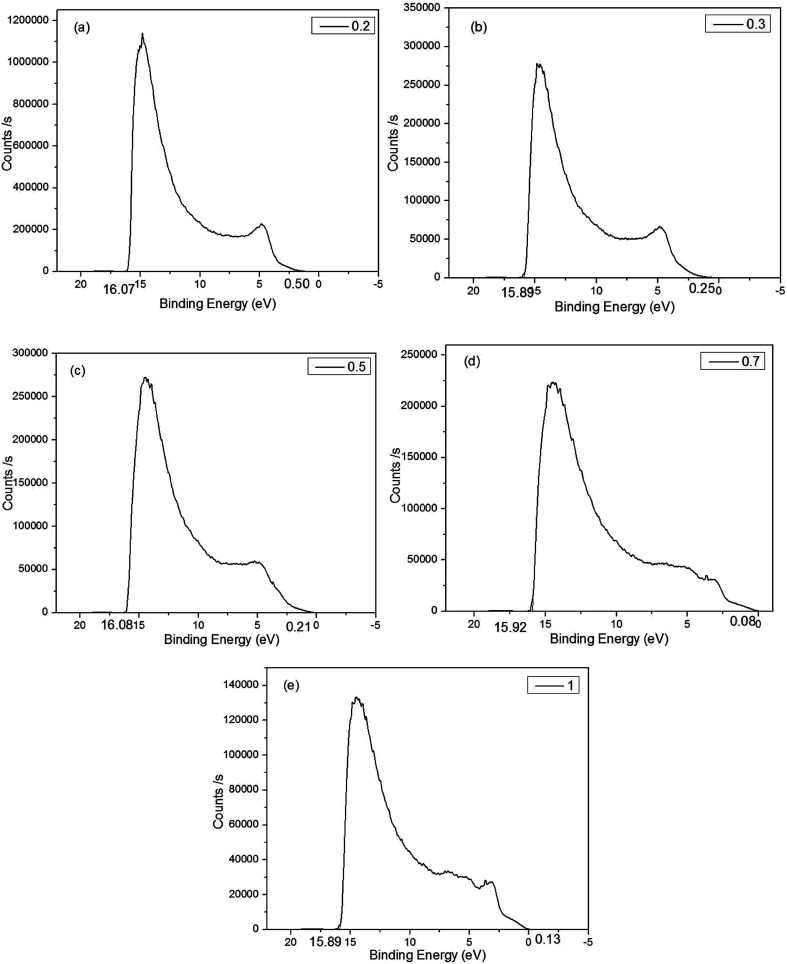
The UPS spectrum of Cu_*x*_Al_1−*x*_S_*y*_ film on FTO glass. (a) *x* = 0.2. (b) *x* = 0.3. (c) *x* = 0.5. (d) *x* = 0.7. (e) *x* = 1.

**Fig. 7:**
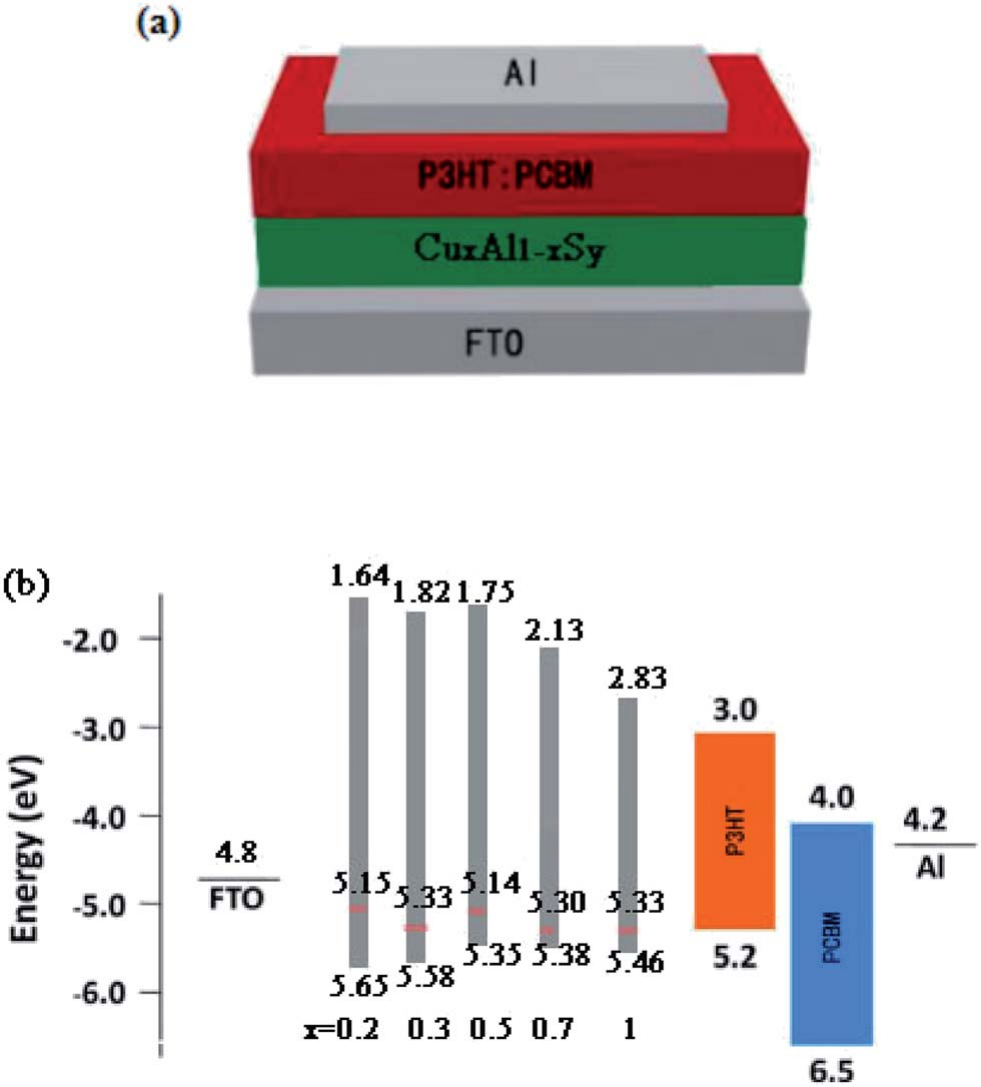
Device structure and energy levels of the OSCs. (a) Schematic of the OSCs. (b) Schematic of the energy alignment of the devices for Cu_*x*_Al_1−*x*_S_*y*_ film (*x* = 0.2, 0.3, 0.5, 0.7, 1).

The UPS measurement was carried out using helium *I*_α_ as the UV source. It can be concluded from Fig. 6 that the binding energies of the Cu_*x*_Al_1−*x*_S_*y*_ films (*x* = 0.2, 0.3, 0.5, 0.7, 1) were 16.07 eV, 15.89 eV, 16.08 eV, 15.92 eV, 15.89 eV, respectively. Thus, the work function of the Cu_*x*_Al_1−*x*_S_*y*_ films (*x* = 0.2, 0.3, 0.5, 0.7, 1) annealed at 150 °C for 10 min was 5.15 eV, 5.33 eV, 5.14 eV, 5.30 eV, 5.33 eV, respectively. Fig. 6 also shows the energy difference between the top of valence band (*E*_V_) and the Fermi level, and it was concluded that the *E*_V_ of the Cu_*x*_Al_1−*x*_S_*y*_ films (*x* = 0.2, 0.3, 0.5, 0.7, 1) were 0.50 eV, 0.25 eV, 0.21 eV, 0.08 eV, 0.13 eV below the Fermi level, respectively, which were 5.65 eV, 5.58 eV, 5.35 eV, 5.38 eV, 5.46 eV, respectively. Take account of the optical band gap of Cu_*x*_Al_1−*x*_S_*y*_ films mentioned previously in Table 1, the bottom of the conduction band (E_A_) is set at 1.64 eV, 1.82 eV, 1.75, eV 2.13 eV, 2.83 eV for *x* = 0.2, 0.3, 0.5, 0.7, 1, respectively. The device structure and energy level alignment are shown in Fig. 7. From the band alignment, the energy band structure of the Cu_*x*_Al_1−*x*_S_*y*_ films (*x* = 0.2, 0.3, 0.5, 0.7, 1) was determined, and the energy band levels obtained for the Cu_*x*_Al_1−*x*_S_*y*_ films with excellent p-type conductivity could be suitable for many opto-electronic devices in the future.

**Fig. 8:**
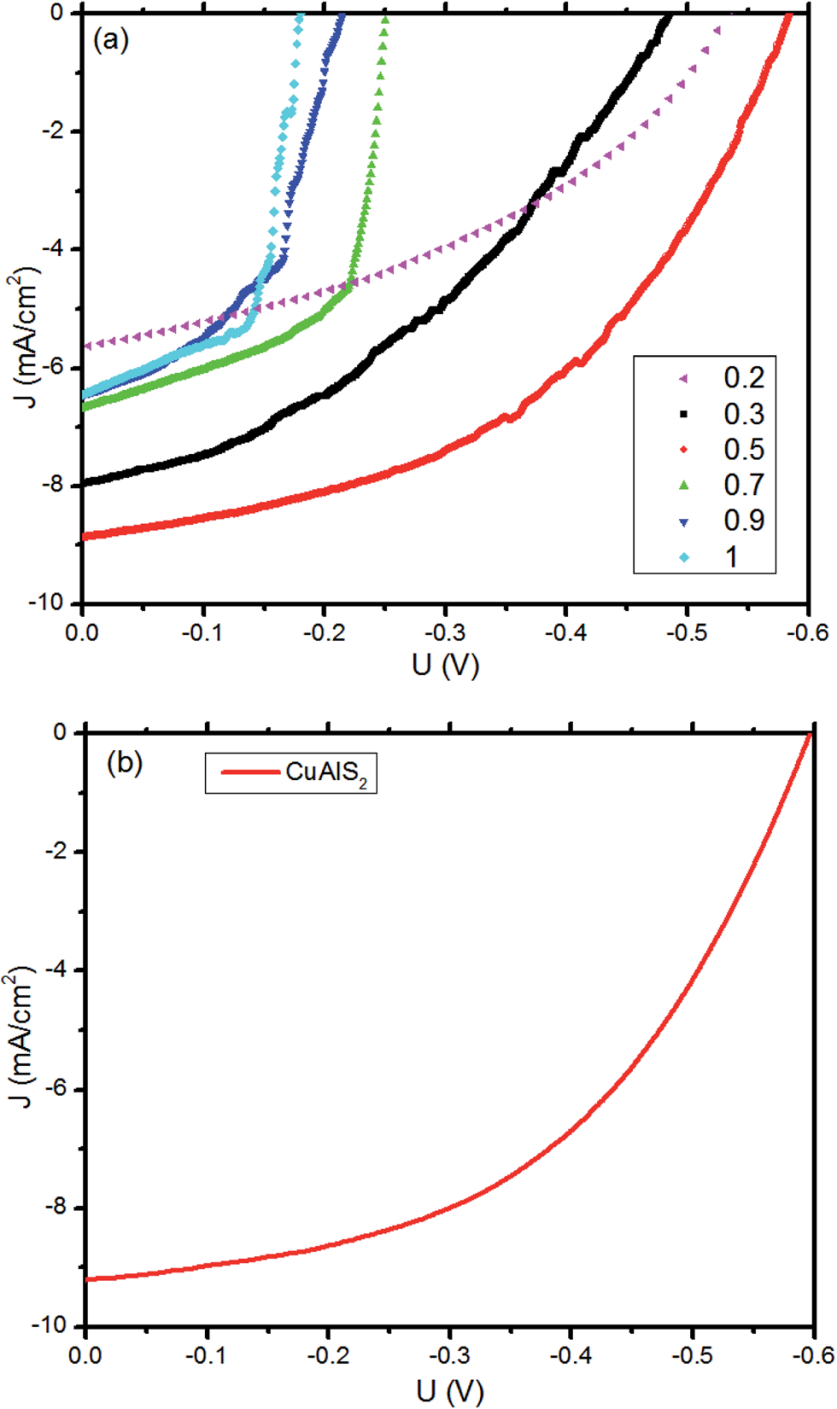
The performance of the devices developed: current density–voltage (*J*–*V*) plots for FTO/HTL/P3HT:PCBM/Al OSCs. (a) Cu_*x*_Al_1−*x*_S_*y*_ as HTL layers. (b) The excellent performance of OSCs using CuAlS_2_ as the HTL layers.

To demonstrate the application of p-Cu_*x*_Al_1−*x*_S_*y*_ films in photovoltaic devices, several heterojunction OSCs were fabricated with the Cu_*x*_Al_1−*x*_S_*y*_ film used as hole transporting layers. The *J*–*V* characteristics, which were measured under standard test conditions (1000 W m^−2^, air mass 1.5 global (AM1.5G) spectrum and 25 °C) for the 0.04 cm^2^ device, are presented in Fig. 8(a). Notably, it was found that a 40 nm CuAlS_2_ film annealed at 150 °C provided superior performance. As summarized in Table 3, for the FTO/Cu_*x*_Al_1−*x*_S_*y*/_P3HT:PCBM/Al devices, the PCE of the OSCs increased from 1.22% to 2.45% when *x* increased from 0.2 to 0.5 because of the increasing hole mobility and the better matched band between Cu_*x*_Al_1−*x*_S_*y*_ and P3HT. However, the PCE decreased while *x* increased from 0.5 to 1, and the reasons for this phenomenon might be the decreasing band gap and the decline of the valence band edge. Compared with CuS film and other Cu_*x*_Al_1−*x*_S_*y*_ films (*x* = 0.7, 0.9) in Fig. 2(a), more light was transmitted by the CuAlS_2_ film with a wider band gap, which allows more use of the incident light under the same conditions. Furthermore, the variation of *E*_V_ is in agreement with the changes in PCE, the *E*_V_ of CuAlS_2_ film is closer to the highest occupied molecular orbital (HOMO) of P3HT compared with other films as shown in Fig. 7(b). Thus, the solar cells using CuAlS_2_ film as HTL produced the highest PCE. Although the energy levels between Cu_*x*_Al_1−*x*_S_*y*_ and P3HT were not sufficiently matched to achieve good hole collection, the variable energy levels of the Cu_*x*_Al_1−*x*_S_*y*_ films with tunable band gap were still attractive, and there will be more research with better matched bands in future. Finally, the best PCE was achieved using CuAlS_2_ film as HTL, and a *J*_sc_ of 9.21 mA cm^−2^ and a *V*_oc_ of 596 mV were observed, delivering a PCE of 2.67%, which is shown in Fig. 8(b). Further comparison with other HTL can be found in Table S1 (ESI†). Therefore, the CuAlS_2_ film is a good material for solar cell applications.

**Table 3:** Summary of the photovoltaic performances of the OSCs^a^ with Cu_x_Al_1-x_S_y_ films used as HTL

HTL (40 nm)	*V*_oc_ (V)	*J*_sc_ (mA cm^−2^)	FF (%)	PCE (%)
0.2	0.535	−5.63	40.39	1.22
0.3	0.533	−7.46	37.93	1.51
0.5	0.585	−8.88	47.16	2.45
0.7	0.251	−6.68	60.82	1.02
0.9	0.215	−6.45	50.05	0.69
1	0.18	−6.45	61.95	0.72
Champion (CuAlS_2_)	0.596	−9.21	48.7	2.67

a There were eight cells for each device in this study. The errors of the PCEs were ±0.10%.

## 4. Conclusion

In summary, the optical and electrical characteristics of the Cu_*x*_Al_1−*x*_S_*y*_ thin films prepared on glass sheets and FTO glass using CBD were studied. Upon varying the precursor ratio of the CBD solution, p-type Cu_*x*_Al_1−*x*_S_*y*_ films with different optical and electrical properties were obtained. The band gap can be adjusted from 2.63 eV to 4.01 eV. In particular, the CuAlS_2_ film had a band gap of 3.60 eV and a hole mobility of 1.00 cm^2^ V^−1^ s^−1^.

Consequently, the films could be used as a hole transporting layer for photocells. The addition of a CuAlS_2_ layer between the anode and the active layer in OSCs can significantly improve the device performance, leading to a 2.67% power efficiency with a device structure of FTO/CuAlS_2_/P3HT:PCBM/Al under simulated AM1.5G 100 mW cm^−2^ illumination. This indicates that CuAlS_2_ is a very promising alternative HTL for OSCs and other opto-electronic devices.

## Conflicts of interest

There are no conflicts to declare.

## Supplementary Material

RA-008-C8RA01299G-s001

## References

[cit1] Zhao D. W., Sexton M., Park H., Baure G., Nino J. C. (2015). High-Efficiency Solution-Processed Planar Perovskite Solar Cells with a Polymer Hole Transport Layer. Adv. Energy Mater..

[cit2] Chang S., Li Q., Xiao X., Wong K. Y., Chen T. (2012). Enhancement of low energy sunlight harvesting in dye-sensitized solar cells using plasmonic gold nanorods. Energy Environ. Sci..

[cit3] Yang Y., Zhou C. H., Xu S., Hu H., Chen B. L. (2008). Improved stability of quasi-solid-state dye-sensitized solar cell based on poly (ethylene oxide)–poly (vinylidene fluoride) polymer-blend electrolytes. J. Power Sources.

[cit4] Wu W. Q., Chen D., Cheng Y. B. (2017). Thin Films of Tin Oxide Nanosheets Used as the Electron Transporting Layer for Improved Performance and Ambient Stability of Perovskite Photovoltaics. Solar RRL.

[cit5] Wu W. Q., Chen D., Li F. (2018). Solution-processed Zn_2_SnO_4_, electron transporting layer for efficient planar perovskite solar cells. Materials Today Energy.

[cit6] Wu W. Q., Feng H. L., Chen H. Y. (2017). Recent advances in hierarchical three-dimensional titanium dioxide nanotree arrays for high-performance solar cells. J. Mater. Chem. A.

[cit7] Chen H. Y., Hou J., Zhang S., Liang Y., Yang G., Li G. (2009). Polymer solar cells with enhanced open-circuit voltage and efficiency. Nat. Photonics.

[cit8] You J. B., Dou L. T., Yoshimura K., Kato T., Ohya K., Moriarty T., Emery K., Chen C. C., Gao J., Li G., Yang Y. (2013). A polymer tandem solar cell with 10.6% power conversion efficiency. Nat. Commun..

[cit9] Chen J., Cui C., Li Y., Zhou L., Ou Q. (2015). Single-Junction Polymer Solar Cells Exceeding 10% Power Conversion Efficiency. Adv. Mater..

[cit10] Zhang C., Zhao D. W., Gu D., Kim H., Ling T., Ultrathin A. (2014). Smooth, and Low-Loss Al-Doped Ag Film and Its Application as a Transparent Electrode in Organic Photovoltaics. Adv. Mater..

[cit11] Kim H. P., Lee H. J., Mohd Yusoff A. R. b., Jang J. (2013). Semi-transparent organic inverted photovoltaic cells with solution processed top electrode. Sol. Energy Mater. Sol. Cells.

[cit12] Krebs F. C. (2009). Roll-to-roll fabrication of monolithic large-area polymer solar cells free from indium-tin-oxide. Sol. Energy Mater. Sol. Cells.

[cit13] Zheng Q., Fang G. J., Bai W. B., Sun N. H., Qin P. L., Fan X., Cheng F., Yuan L. Y., Zhao X. Z. (2011). Efficiency improvement in organic solar cells by inserting a discotic liquid crystal. Sol. Energy Mater. Sol. Cells.

[cit14] Lim F. J., Ananthanarayanan K., Luther J. (2012). Influence of a novel fluorosurfactant modified PEDOT: PSS hole transport layer on the performance of inverted organic solar cells. J. Mater. Chem..

[cit15] Norrman K., Madsen M. V., Gevorgyan S. A., Krebs F. C. (2010). Degradation Patterns in Water and Oxygen of an Inverted Polymer Solar Cell. J. Am. Chem. Soc..

[cit16] Cheng F., Fang G. J., Fan X., Huang H. H., Zheng Q., Qin P. L., Lei H. W., Li Y. F. (2013). Enhancing the performance of P3HT: ICBA based polymer solar cells using LiF as electron collecting buffer layer and UV–ozone treated MoO_3_ as hole collecting buffer layer. Sol. Energy Mater. Sol. Cells.

[cit17] Cheng F., Fang G., Fan X., Liu N., Sun N., Qin P. (2011). Enhancing the short-circuit current and efficiency of organic solar cells using MoO and CuPc as buffer layers. Sol. Energy Mater. Sol. Cells.

[cit18] Bernède J. C., Cattin L., Makha M., Jeux V., Leriche P., Roncali J., Froger V., Morsli M., Addou M. (2013). MoO_3_/CuI hybrid buffer layer for the optimization of organic solar cells based on a donor–acceptor triphenylamine. Sol. Energy Mater. Sol. Cells.

[cit19] Sun N., Fang G., Qin P., Zheng Q., Wang M., Fan X., Cheng F., Wan J., Zhao X., Liu J., Carroll D. L., Ye J. (2010). Efficient flexible organic solar cells with room temperature sputtered and highly conductive NiO as hole-transporting layer. J. Phys. D: Appl. Phys..

[cit20] Sun N., Fang G., Qin P., Zheng Q., Wang M., Fan X. (2010). Bulk heterojunction solar cells with NiO hole transporting layer based on AZO anode. Sol. Energy Mater. Sol. Cells.

[cit21] Wang Y. H., Zhang Q., Li G. F., Shi Z., Tan H. (2008). Growth and Properties of P-Type Zn-Doped CuAlS_2_ Films. Chin. J. Vac. Sci. Technol..

[cit22] Poulose A. C., Veeranarayanan S., Aravind A., Nagaoka Y., Yoshida Y. (2012). Synthesis of CuAlS_2_ Nanocrystals and Their Application in Bio-Imaging. Mater. Express.

[cit23] Ghosh B., Pradhan S. K. (2011). One-step fastest method of nanocrystalline CuAlS_2_ chalcopyrite synthesis, and its nanostructure characterization. J. Nanopart. Res..

[cit24] Chichibu S., Shishikura M., Ino J. (1991). Electrical and optical properties of CuAlSe_2_ grown by iodine chemical vapor transport. J. Appl. Phys..

[cit25] Huang F. Q., Liu M. L., Yang C. (2011). Highly enhanced p-type electrical conduction in wide band gap Cu_1+*x*_Al_1−*x*_S_2_ polycrystals. Sol. Energy Mater. Sol. Cells.

[cit26] Vahidshad Y. (2012). Synthesis and Characterization of CuAlS_2_ Nanoparticles by Facile Heat Arrested Method. Phys. E.

[cit27] Caglar M., Ilican S., Caglar Y. (2008). Structural, morphological and optical properties of CuAlS_2_ films deposited by spray pyrolysis method. Opt. Commun..

[cit28] Alwan T. J., Jabbar M. A. (2014). Structure and optical properties of CuAlS_2_ thin films prepared *via* chemical bath deposition. Turk. J. Phys..

[cit29] Lei H., Fang G., Cheng F., Ke W., Qin P., Song Z. (2014). Enhanced Efficiency in Organic Solar Cells *via in situ* Fabricated p-type Copper Sulfide as the Hole Transporting Layer. Sol. Energy Mater. Sol. Cells.

[cit30] Lei H., Qin P., Ke W., Guo Y., Dai X., Chen Z., Wang H. (2015). Performance enhancement of polymer solar cells with high work function CuS modified ITO as anodes. Org. Electron..

[cit31] Ke W., Fang G., Lei H., Qin P., Tao H., Zeng W. (2014). An efficient and transparent copper sulfide nanosheet film counter electrode for bifacial quantum dot-sensitized solar cells. J. Power Sources.

[cit32] Tao C., Ruan S., Xie G., Kong X., Shen L., Meng F., Liu C., Zhang X., Dong W., Chen W. (2009). Role of tungsten oxide in
inverted polymer solar cells. Appl. Phys. Lett..

[cit33] Liao X. H., Chen N. Y., Xu S., Yang S. B., Zhu J. J. (2003). A microwave assisted heating method for the preparation of copper sulfide nanorods,. J. Cryst. Growth.

[cit34] Arai T., Horiguchi M., Yanagida M., Gunji T. (2009). Reaction Mechanism and Activity of WO_3_-Catalyzed Photodegradation of Organic Substances Promoted by a CuO catalyst. J. Phys. Chem. C.

