# Supplementary material for: A simple synthesis of transparent and highly conducting p-type CuxAl1−xSy nanocomposite thin films as the hole transporting layer for organic solar cells
Source: RSC Adv. 2018 May 8;8(30):16887–96. doi: 10.1039/c8ra01299g (PMC9080296; doi:10.1039/c8ra01299g)
Supplement: RA-008-C8RA01299G-s001 [file RA-008-C8RA01299G-s001.pdf]

Electronic Supplementary Material (ESI) for RSC Advances.
This journal is © The Royal Society of Chemistry 2018

Supporting information

Facile Synthesis of Transparent and Highly Conducting *p*-type $Cu_xAl_{1-x}S_y$ Nanocomposite Thin Films as the Hole Transporting Layer for Organic Solar Cells

Xin Dai[a,b], Hongwei Lei[a], Cong Chen[a], Yaxiong Guo[a], Guojia Fang*[a,b]

[a] *Key Laboratory of Artificial Micro- and Nano-structures of Ministry of Education of China, School of Physics and Technology, Wuhan University, Wuhan 430072, PR China*

[b] *Shenzhen Institute of Wuhan University, Shenzhen 518055, PR China*

**E-mail: gjfang@whu.edu.cn ; Tel: +86 (0)27 87642784 ; Fax: +86 (0)27 68752569*

Table S1. Comparing the major performance of the solar cells using P3HT:PCBM as the active layer.

HTL	Deposition method	Voc (V)	Jsc (mA/cm^2)	FF (%)	PCE (%)	ETL	reference
CuS	Magnetron sputtering and Hydrothermal method	0.55	-10.96	56	3.4	LiF	[1]
PEDOT:PSS	Spin coating	0.56	-9.2	59	3.0	LiF	[1]
V_2O_5	Thermally evaporated	0.59	-8.83	59.1	3.10	Ca	[2]
MoO_3		0.60	-8.94	61.9	3.33	Ca	[2]
Cr_2O_3	Magnetron sputtering	0.53	-9.98	58.6	3.05	None	[3]
MoS_2	Spin coating and thermolysis	0.60	-7.81	63	2.96	LiF	[4]

WS_2	at 900°C	0.61	-7.87	64	3.08	LiF	[4]
WS_2	ultra-sonication and spin-coating	0.49	-8.06	61	2.40	LiF	[5]
$CuAlS_2$	Chemical bath deposition	0.60	-9.21	48.7	2.67	None	Present work

For P3HT:PCBM system, the efficiency is usually in the range of 2.5%~3.5% according to literature reports, as seen in Table S1. Here, we reported a new promising hole transporting layer (HTL) with tunable band gap. Comparing with other methods above, chemical bath deposition (CBD) is very facile and safe which only needs very low temperature and a heating equipment. However, other materials require either high temperature or complex vacuum system for deposition. For instance, metal sulfides like MoS_2 and WS_2 need a rather high temperature for thermolysis in H_2, which is very dangerous. The CuS film mentioned in reference 1 has a 2D sheet-like morphology which is very different from $Cu_xAl_{1-x}S_y$ thin films and a better matched band in the solar cells, resulting a higher efficiency. However, the CuS film deposited by magnetron sputtering and hydrothermal method requires a complex vacuum system. Thus, $Cu_xAl_{1-x}S_y$ thin films with tunable band gap would be very charming owing to convenient deposition method and their excellent properties. When comparing with the CuS film deposited by CBD, $CuAlS_2$ film has a wider band gap and a higher valence band edge that better matches with P3HT in organic solar cell, leading to better performance. The efficiency seems not very high at this moment, which may be caused by the rough surface morphology of $Cu_xAl_{1-x}S_y$ films and the mismatched energy level between $Cu_xAl_{1-x}S_y$ and P3HT. We will focus on improving

the PCE in our further work.

References

[1] H. Lei, G. Fang, F. Cheng, W. Ke, P. Qin, Z. Song, Enhanced Efficiency in Organic Solar Cells via in situ Fabricated p-type Copper Sulfide as the Hole Transporting Layer, Solar Energy Materials & Solar Cells, 2014, 128, 77-84.

[2] V. Shrotriya, G. Li, Y. Yao, Transition metal oxides as the buffer layer for polymer photovoltaic cells, Applied Physics Letters, 2006, 88(7), 073508-073508-3.

[3] F. Gilles, A.Y. Goy, P. Shue, A. Zelenetz, Composition-dependent phase separation effects of organic solar cells using P3HT:PCBM as active layer and chromium oxide as hole transporting layer, Applied Surface Science, 2011 , 257 (9) :3952-3958.

[4] K.C. Kwon, C. Kim, Q.V. Le, S. Gim, J.M. Jeon, Synthesis of Atomically Thin Transition Metal Disulfides for Charge Transport Layers in Optoelectronic Devices, Acs Nano, 2015, 9 (4), 4146-55.

[5] Q.V. Le, T.P. Nguyen, S.Y. Kim, UV/ozone-treated WS_2 hole-extraction layer in organic photovoltaic cells, physica status solidi (RRL) - Rapid Research Letters, 2014, 8(5),390-394.
